# Considerations and perspectives on phage therapy from the transatlantic taskforce on antimicrobial resistance

**DOI:** 10.1038/s41467-025-64608-3

**Published:** 2025-12-04

**Authors:** Kyung Moon, Carmen Coxon, Christine Årdal, Radu Botgros, Sarah Djebara, Laura Durno, Cara R. Fiore, Jean-Baptiste Perrin, Dennis M. Dixon, Marco Cavaleri

**Affiliations:** 1 National Institute of Allergy and Infectious Diseases, National Institutes of Health, Bethesda, MD USA; 2 MHRA Science Campus, Blanche Lane, South Mimms, Hertfordshire, UK; 3 Norwegian Institute of Public Health, Oslo, Norway; 4 Public Health Threats Department, European Medicines Agency, Amsterdam, the Netherlands; 5 Center for Infectious diseases ID4C, Queen Astrid military hospital, Brussels, Belgium; 6 Health Products and Food Branch, Health Canada, Ottawa, Canada; 7 Center for Biologics Evaluation and Research, the U S Food and Drug Administration (US FDA), Silver Spring, MD USA; 8 European Commission, Health emergency preparedness and response authority (HERA), Brussels, Belgium

**Keywords:** Infectious diseases, Biologics

## Abstract

Amid rising antimicrobial resistance and limited antibiotic innovation, bacteriophages are gaining attention as potential therapeutics across human health, animal, and food sectors. Despite historical use, their clinical application in humans remains constrained by scientific, industrial, and regulatory challenges. To address these issues, the Transatlantic Task Force on Antimicrobial Resistance (TATFAR) convened sessions with experts from member parties. This perspective synthesizes insights from the TATFAR group, highlighting regulatory differences, research gaps, and opportunities for international collaborations to advance bacteriophage therapy.

## Abstract (web summary)

Phage therapy shows promise for treating bacterial infections, including those resistant to antibiotics, but its development faces scientific and regulatory hurdles. This Perspective outlines the regulatory landscape in TATFAR member states and their collaborative effort to advance phage therapy.

## Introduction

A 2022 report estimated that there were approximately 4.95 million deaths worldwide in 2019 associated with Antimicrobial Resistance (AMR), of which 1.27 million were directly linked^1^. Projections suggest this number could escalate to 8.22 million deaths globally in 2050^2,3^, alongside an economic burden of $100 trillion^4^, if effective solutions are not implemented.

In response to the growing threat of AMR and the limitations of traditional antibiotics, bacteriophages (phages) are being reconsidered as an alternative or complementary therapy for bacterial infections. These natural viruses selectively target and eradicate bacteria, presenting several potential advantages, such as preservation of the microbiota, while generally demonstrating a favourable safety profile with minimal known adverse events^5,6^. Notably, phages have shown promise in treating infections caused by multidrug-resistant bacteria or other difficult-to-treat infections that are challenging for conventional antibiotics^7,8^. Phage therapy, which has been utilised notably in Russia, Georgia, and Poland for over a century, has gained renewed global attention in the last decade^9^. This is reflected in the formation of various phage consortia and initiatives by several countries aimed at improving patient access to phage therapy^10–13^. However, the benefit-risk balance of phage therapy medicinal products intended for clinical use remains uncertain, primarily due to insufficient efficacy data from randomised controlled clinical trials (RCTs). Most clinical data published in the medical literature are stemming from case reports, case series, or small uncontrolled studies, which are susceptible to publication bias and often yield inconclusive outcomes regarding efficacy^14,15^. The numerous RCTs conducted to date have largely failed to demonstrate efficacy, although a few promising data have recently emerged^16–18^. This further underscores the need for rigorous clinical development and evaluation^19,20^.

Established in 2009 following the European Union (EU) – United States (US) Summit Declaration, the Transatlantic Task Force on Antimicrobial Resistance (TATFAR) collaborates and exchanges best practices to strengthen domestic and global efforts to combat AMR. Through its 2021–2026 work plan, TATFAR has formed a dedicated working group to support the development of new antimicrobials, alternative approaches, and diagnostic devices by facilitating the exchange of regulatory and scientific information^21^. In November 2023, TATFAR convened an in-person meeting with phage experts, public health officials, and regulatory authorities from Canada, the EU, Norway, the United Kingdom (UK), and the US, as well as from other partner countries^22^. The meeting focused on addressing critical issues related to phage therapy, featuring presentations on two distinct developments. The first involves a standardised (pre-defined) finished medicinal product that consists of one or more phage strains. In contrast, the second, more personalised approach selects active phages for treatment from a pre-existing phage collection, tailoring the product to individual patients^23^. In addition, the meeting explored phage applications in animal health, particularly in the context of salmon production. These presentations provided illustrative examples intended to facilitate discussion regarding regulatory challenges and frameworks within TATFAR countries. Participants engaged in a comprehensive discussion on access to phage therapy, the status of phage clinical trials, obstacles to clinical trial initiation, and existing regulatory requirements across the TATFAR member countries.

This article synthesises insights from the TATFAR meeting and subsequent consideration and provides an overview of the regulatory landscape for phage therapeutics among the TATFAR member countries. It also reflects on the roles of these countries in the global effort to support phage therapy.

## Phage therapy strategies & clinical trial challenges

Interest in phage therapy has led to a recent increase in the number of clinical trials across TATFAR member states. These trials include some RCTs, but the majority are smaller studies which are not suitable for regulatory approval purposes. As of February 2025, over 60 interventional phage studies are listed on ClinicalTrials.gov as either completed, actively recruiting, or planned.

In the absence of licensed products, several countries, including Belgium, Georgia, Poland, Portugal, France, and the US, have initiated strategies to offer unlicensed personalised phage therapy to patients in need^10,24^. These efforts are primarily guided by Article 37 of the Declaration of Helsinki^25^, which addresses unproven clinical practice interventions.

The following section outlines two different strategies for clinical use of phage therapy: the Belgian magistral pathway, exemplifying personalised treatment, and the Antibacterial Resistance Leadership Group (ARLG)’s PHAGE, illustrating RCTs involving fixed phage medicinal products.

### Belgian magistral phage strategy

The Belgian magistral phage strategy^26^, established in 2016, is a national initiative designed to provide personalised phage therapy (not approved as a medicinal product) for patients with infections caused by AMR pathogens that have limited or no treatment options. This strategy was developed through extensive multisectoral efforts, led by the team at the Queen Astrid Military Hospital under the Belgium Ministry of Defence.

Phage preparations administered under this strategy are not manufactured under the Good Manufacturing Practice (GMP) framework. The Queen Astrid Military Hospital published results of a multicenter, multinational, retrospective observational study involving 100 difficult-to-treat infections (BT100)^10^. These cases were treated with 26 phages and 6 cocktails, including two commercially available cocktail products from the Eliava Institute in Georgia. In certain cases, pre-adaptation was necessary. According to the authors, the use of phages in conjunction with antibiotics appears to be associated with a higher likelihood of successful eradication of the infections, although the chosen study design does not allow for robust conclusions to be made.

This strategy does not generate the type of evidence produced by RCTs, which is essential for demonstrating the efficacy of phage therapy. Although capturing the benefits of personalised phage therapies necessitates more complex study designs than for ready-to-use phage(s), the value of personalised phage therapy needs to be demonstrated by RCTs^11,27–36^. In addition, the implementation of this strategy has revealed some logistical challenges related to the production and delivery of personalised phage products. These challenges will need to be addressed to ensure broad and sustainable access to personalised therapy, should the proof-of-concept on efficacy be demonstrated.

### ARLG’s PHAGE

The National Institute of Allergy and Infectious Diseases (NIAID) at the US National Institutes of Health (NIH) has supported multiple programmes facilitating phage research via multifaceted funding mechanisms. Notably, the Antibacterial Resistance Leadership Group (ARLG), a stand-alone AMR clinical network funded by NIAID, has launched the PHAGE trial. This phase 1b/2 randomised, double-blind, placebo-controlled trial investigates a single dose of intravenous (IV) phage therapy in clinically stable subjects with cystic fibrosis who are chronically colonised with *Pseudomonas aeruginosa*. The investigational phage product, WRAIR-PAM-CF1, comprises four anti-pseudomonal phages in equal proportions (1:1:1:1). The primary objectives of the study are to assess the safety profile of WRAIR-PAM-CF1 and to evaluate its microbiological activity^37^.

The trial, which consists of three stages^37^, has completed subject enrolment for all stages. However, several challenges merged during the process, particularly in developing protocols for laboratory assay and clinical studies related to phage investigational new drugs (IND). To address these issues, the study team collaborated closely with NIH and the FDA to create a comprehensive study design and protocol. This collaboration included identifying a targeted subject group for phage susceptibility testing and understanding the regulatory pathway necessary to effectively evaluate the therapy in a clinical setting.

Another significant challenge was finding manufacturers capable of producing phage products on a large scale. In addition, ensuring the adequate stability of these products during production proved to be problematic. Patient recruitment was also complicated by narrow eligibility criteria. For instance, one inclusion criterion required that subjects not be on antibiotics at the time of enrolment and that they produce a minimum of 2 ml of sputum over a 30 min period for laboratory testing. This sputum production requirement was particularly challenging for patients receiving new cystic fibrosis drugs (e.g., Trikafta^®^)^38^, which specifically reduced sputum production^39,40^.

To enhance subject enrolment, the NIH sought collaboration with international colleagues to extend sites abroad; however, these efforts did not result in the establishment of international sites. Nevertheless, the NIH positioned itself as a key stakeholder in advancing the field. Drawing from the insights gained during the trial’s launch, the ARLG team, in concert with the NIH, has published recommendations to guide clinicians in considering experimental phage therapy for treating subjects in clinical practices^41^.

## Veterinary use of phage products

Despite nearly two decades of application in the food industry, phage products have not yet been licensed as veterinary medicinal products. Phages are being investigated for their potential to control food-borne pathogens that pose a threat to human health, such as *Listeria*, *Salmonella*, and *Shiga* toxin-producing *Escherichia coli* (*E. coli*). In addition, research has explored phage effectiveness against pathogens that cause bovine mastitis in dairy cows, such as *E. coli* and *Staphylococcus aureus*^42^. Phage therapy has also been studied for treating skin infections in companion animals, such as dogs and cats, particularly in Europe and Japan^43^. However, these applications remain mainly experimental or in the early stages of commercialisation, with no licensed products available in most parts of the world. The following section outlines an example from aquaculture presented during the in-person TATFAR meeting.

### Use of phages in Norwegian salmon farms

In Norway, phages are used as a biocontrol product in farmed salmon. Norwegian farmed salmon are individually vaccinated against various pathogens, enabling large-scale salmon production with minimal or no use of antibiotics^44^. During vaccination, young fish are more susceptible to infection caused by *Yersinia ruckeri*, which can arise due to the stress of the vaccination process^45^. To mitigate this risk, phage therapy is employed by adding phages to tanks of water through which young salmon swim prior to vaccination, thereby minimising the likelihood of infection. As the phage treats the water rather than the fish directly, it is classified as a biocontrol product and is not regulated by the EMA veterinary medicines guidelines published in 2023: *Guideline on quality, safety and efficacy of veterinary medicinal products specifically designed for phage therapy*^46^. As natural products, phages increase their concentration locally but constitute a fraction of the total phage population in the water, degrading in the absence of a host.

ACD Pharma has developed this phage biocontrol product in collaboration with Norwegian salmon companies. The phage is specifically tailored to Norwegian salmon and the associated strain of *Yersinia ruckeri* that affects them. The same phage seems ineffective in preventing infections in Norwegian farmed rainbow trout, or Chilean farmed salmon, indicating that geographic adaptation is necessary for efficacy in different environments^47^.

Phages are also seen as a potential solution for other bacterial infections in aquaculture, such as the Gram-negative bacteria *Moritella viscosa*, which causes winter sores in salmon. Given that the phage would act directly on the fish, it must be classified as a medicinal product and, consequently, comply with the EMA’s new guidelines.

The EMA’s guideline acknowledges that the market for each phage is likely to be small due to its activity being restricted to specific bacterial strains. Frequent adjustments to phage composition may be necessary as bacterial strains evolve. The development of these products may face challenges related to intellectual property rights, like patents, which may not apply due to the natural ubiquity of phages. Even if patents are applicable, the economic feasibility of pursuing them is uncertain, given the need to modify compositions in response to bacterial evolutionary changes. A competitive advantage lies in the close collaboration between the salmon industry and the phage manufacturer to develop tailored phages suited to the particular environment. However, the costs associated with required trials may exceed potential revenues, potentially hindering innovation in this promising area of phage application.

## Regulatory landscape for human phage therapy in TATFAR regions

As interest in phage therapy continues to grow, various regulatory approaches may be used in different regions around the world. In the following sections, we summarise the regulatory frameworks related to the approval and to the unlicensed use of phage therapy within four TATFAR regions. This includes the legal definition of phage-containing medicinal products, the regulatory expectations for approval, specific considerations for personalised phage products, and access to unlicensed products. A summary that may be valuable for researchers, healthcare providers, and industry professionals engaged in phage research and development is outlined in Table 1.

**Table 1:** Overview of regulatory requirements for phage therapy among TATFAR member countries

Agency	EU EMA	Health Canada	UK MHRA	US FDA
Legal classification	• Phages are considered as biological medicinal products. • Engineered phages can be considered as ATMPs.	• Natural and recombinant phage are considered as biological drugs.	• Naturally occurring phages are biological medicinal products. • Engineered phages will be biologics or GTMPs depending on the modification.	• PTPs are biological products when proposed to be used to cure, treat, mitigate or prevent diseases in humans
Current access	• No phage products have received central market authorisation; some Member States have national authorisations granted prior to their accession to the EU. • Individual patients may access phage therapy products via compassionate use during clinical trials or expanded access when a marketing authorisation application is submitted to a national competent authority. • Approaches vary by EU Member states.	• No phage products have market authorisation • Phage therapy is available only as an experimental drug through a clinical trial, either single patient or larger clinical trials.	• No phage products have market authorisation. • Phages are used as unlicensed medicinal products.	• No PTPs are licensed for use in humans. • An IND application is required if an unlicensed product, or a licensed product proposed for an unlicensed use, is intended to cure, treat, mitigate or prevent a disease in humans. • Expanded Access is available if regulatory criteria are met^91^.
Anticipated pathways for market authorisation	• There is no specific framework for phage therapy • For a pre-established combination of phages, the EMA expects RCTs that demonstrate superiority of the candidate phage product in addition to the BAT, compared to BAT alone. • Existing regulatory tools allow for periodical updates of phage cocktails. (e.g., variation, extension of indications) • For personalised phage products, there is currently no defined framework to customise regulatory requirements. This issue is under consideration in the ongoing revision of EU pharmaceutical legislation.	• Existing regulatory framework is applicable. • A new agile licensing framework (ATPathway)^70^ for innovative health products is available, allowing Health Canada to customise regulatory requirements for phage therapy, if needed.	• Existing regulatory framework is applicable. GM medicines require evaluation by the Health and Safety Executive (HSE) and/or the Department for the Environment, Food, and Rural Affairs (Defra).	• Existing regulatory framework is applicable.
Process for clinical trial application and approval	• A CTA is required for clinical trials. • Investigational phage products can be accessed via single-patient phage use, compassionate use or expanded access programmes via Member States clearance.	• A CTA is required for individual patient access or for larger clinical trials. • Currently, no emergency use mechanisms	• Clinical trials require approval from the regulator (safety) and the NHS (ethics). • MHRA is currently overhauling its clinical trial approval process and will introduce a new streamlined framework soon.	• An IND is required if an unlicensed product, or a licensed product proposed for an unlicensed use, is intended to cure, treat, mitigate or prevent a disease in humans^102^.
Guidance and scientific advice	• The EMA recommends all developers engage with the EU regulatory network as early as possible to discuss their programmes thoroughly and receive appropriate guidance.	• HC recommends all developers to engage with HC as early as possible. Clinical trial sponsors can contact Health Canada’s BRDD for specific guidance and to request a formal meeting.	• The MHRA has published all regulatory guidance pertaining to phage therapeutic products. • The MHRA has published a manuscript in consultation with the Innovate UK Phage Innovation Network to help interpret the regulatory guidance. • Developers can seek advice and ask questions from the MHRA^103^.	• The FDA recommends that once a specific product is identified and a clinical plan proposed, developers should approach the OVRR/CBER to request a formal meeting^104^.
Anticipated preclinical study requirement (PK/PD, stability, toxicity)	• Nonclinical studies should provide information on PK/PD and investigate the potential for emergence of resistance and impact on the gut microbiota. • Genotoxicity, carcinogenicity studies, and reproductive toxicity are generally not required.	• Nonclinical studies may inform clinical development. Generally, GLP toxicity studies would normally not be required. • Product developers should contact BRDD.	• It is likely preclinical data packages will align with expectations for biological medicines as outlined in ICH guidance documents^73^; some engineered phage products will be classified as gene therapy medicinal products - advice should be sought from the MHRA if clarity on evidence requirements is needed.	• Nonclinical studies may inform clinical development. Generally, GLP toxicity studies may not be required for phage therapy products^85,105^. Product developers should contact the agency prior to conducting GLP studies.^a^
Manufacturing requirements: GMP, quality standards	• The manufacturing of the final drug product should be GMP-compliant. • The manufacturing process should be standardised, including description, validation, and control. • Phages must be identified, and their purity, potency, and viability must be maintained	• For RCTs, GMP compliance is not required, but the expectation for “GMP-like” conditions will increase throughout clinical development to ensure consistency in phage manufacturing, quality control, and thereby enhancing the robustness of clinical trial results. • For single-patient CTAs, Health Canada has developed draft guidance on key quality attributes (unpublished; available upon request). Health Canada may request further phage characterisation and details on manufacturing processes and controls	• Unlicensed medicines must be manufactured in accordance with GMP requirements. Examples include ‘specials’, manufacturing licences, and IMP licences. • Compliance with local healthcare regulations may be required for the use of phages, depending on the type of product and its use (licensed/unlicensed).	• Manufacturing and process controls should be appropriate for the current clinical phase of the investigational phage therapy product development.
Sourcing and management of phages and phage banks	• Phage banks need to be established in accordance with ICHQ5D.	• Phages used in single-patient trials can be sourced from existing phage banks inside or outside of Canada. • The government of Canada is developing a centralised phage collection and susceptibility testing service to provide phages domestically for treating cases driven by AMR^61^.	• Unlicensed medicines must be produced under GMP. • There are currently no GMP-compliant phage production sites in the UK. • There are no licensed phage products in the UK at the time of writing.	• Relevant and product-specific characterisation information about phages should be included for all phages included in the investigations. • Manufacturing process (including cell banking) and stability of investigational products should be appropriate for the current clinical stage of the phage therapy product development^84^.
Environmental risk assessment	• Environmental Assessment information is required.	• Environmental Assessment information is required (New Substances Notification)^106^	• Environmental impact assessment is required by HSE and Defra (see above).	• Environmental Assessment information is required^107^.

^a^GLP toxicities studies include repeat dose, Genotoxicity, carcinogenicity studies, and reproductive GLP toxicity studies.

*ATMPs* Advanced Therapy Medicinal Products, *ATPathway* Advanced Therapeutic Products Pathway, *BAT* Best Available Therapy, *BRDD* Biologic and Radiopharmaceutical Drugs Directorate, *CBER* Centre for Biologics Evaluation and Research, *cMAA* Certified Medical Administrative Assistant, *CTA* Clinical Trial Application, *DEFRA* Department for Environment, Food & Rural Affairs, *EMA* European Medicines Agency, *EU* European Union, *FDA* Food and Drug Administration, *GLP* Good Laboratory Practice, *GMP* Good Manufacturing Practices, *GTMPs* Gene therapy based medicinal products, *HC* Health Canada, *HSE* Health and Safety Executive, *IMP* Investigational Medicinal Products, *IND* Investigational New Drug, *ICHQ5D* the International Council for Harmonisation of Technical Requirements for Pharmaceuticals for Human Use quality guideline Q5D, *MHRA* Medicines and Healthcare Products Regulatory Agency, *NHS* National Health Service, *OVRR* Office of Vaccines Research & Review, *PK/PD* Pharmacokinetics-Pharmacodynamics, *PTPs* Phage Therapy Products, *RCT* Randomised controlled Clinical Trials.

### European Union (European Medicines Agency)

In the EU, phages used for treatment of infections are classified as biological substances, and phage-containing medicines are considered as biological medicinal products under the existing EU pharmaceutical legislation (Directive 2001/83/EC)^48^. The manufacturer must characterise the biological properties of each phage, including its lytic activity. For phages, the centralised authorisation procedure is generally used^49^. Developers submit a single marketing authorisation application to the European Medicines Agency (EMA). This is evaluated by the EMA’s Committee for Medicinal Products for Human Use (CHMP). Based on a positive CHMP recommendation, the European Commission (EC) issues a legally binding decision to authorise the phage-containing medicinal products. Once granted, this marketing authorisation is valid in all EU and European Economic Area (EEA) countries.

- **Regulatory considerations**

Developers are advised to consult all relevant EMA guidelines aimed at developing medicines for the prevention and treatment of bacterial infections^50,51^. As with every biological product seeking approval, phage therapy medicinal products need to adhere to specific quality parameters. Both individual phages and the final preparation (as drug substance and drug product, respectively) should follow a standardised manufacturing process that includes a clear description, validation, and control. Phage banks need to be established in accordance with the International Council for Harmonisation of Technical Requirements for Pharmaceuticals for Human Use (ICH) quality guideline Q5D, which covers the derivation and characterisation of cell substrates used for the production of biotechnological/biological products (ICHQ5D)^52^. Phages should be properly identified, and their purity, potency, and viability must be maintained. In addition, a host bacterial cell bank must be established, ensuring that hosts are screened for the absence of resistance to antibiotics, virulence factors, and prophages. The manufacturing of the final drug product should comply with GMP^23,53^.

Nonclinical studies should gather proof-of-concept data on the intended use of the medicine, determining whether the medicine will be used in conjunction with the best available therapy (BAT) with antibiotics or as a stand-alone therapy. These studies should also inform pharmacokinetics (PK) of the disposition of phages inside the body and support the chosen dosage regimen (in vivo Pharmacodynamics [PD]). It may be beneficial to investigate the potential emergence of resistance and its impact on gut microbiota. Nonclinical development discussions with EU regulators via scientific advice are recommended, although toxicological requirements might be abbreviated when relevant animal models are utilised. Typically, genotoxicity, carcinogenicity, and reproductive toxicity studies may not be required. As previously emphasised, it is crucial to obtain evidence of efficacy and safety through well-designed RCTs. Most of these trials will likely compare the candidate phage medicinal product in combination with the BAT against BAT alone. In this case, the EMA may require demonstrating the superiority of the combination therapy over the BAT for approval. However, alternative study designs for certain types of infection could be discussed with EMA. This primarily applies to pre-established combinations of phages administered topically or systemically in the form of a “phage cocktail.” Relevant clinical endpoints, such as time to cure or relapse rates, and other clinical benefits, should be agreed upon upfront with the EU regulators. To maintain therapeutic efficacy, these products will likely require periodical updates. Existing regulatory tools may need to be refined to facilitate the timely adjustment of the phage therapy medicinal products. In addition to efficacy data, safety data need to be collected to reassure European regulators of the safety of these products. Potential risks, such as endotoxemia from bacterial lysis, anaphylactic shock, and transduction of antibiotic resistance genes by phages, cannot be excluded a priori.

Candidate medicines based on engineered phages could be classified as advanced therapy medicinal products (ATMPs). The extent of data on quality, safety, and efficacy will be determined based on a risk-based approach, in accordance with existing EU legislation. In certain situations, such medicines also need to comply with the Directive regulating the release of genetically modified organisms^53,54^.

The EU has been implementing various initiatives to clarify the regulatory framework for phage therapy, particularly pertaining to the development and manufacturing of phage products^55^. In March 2024, the European Pharmacopoeia Commission (EPC) adopted a new general chapter on Phage therapy Medicinal Products (5.31)^56^, which outlines the requirements for the production and control of these products. This chapter allows for a degree of flexibility that corresponds to the complex approaches currently employed in this rapidly evolving field. In addition, in December 2023, EMA released a concept Paper addressing ‘the Establishment of a Guideline on the Development and Manufacture of Human Medicinal Products Specifically Designed for Phage Therapy,’ which underscores the existing gap in appropriate regulatory guidance for phage medicinal products intended for human use in the EU^23^.

- **Specific aspects applying to personalised phage products**

The development of personalised phage products involves a more complex scenario. In these cases, it is essential to establish the combination of phages, either trained on the actual bacteria causing the infection or taken from a phage bank, through a process known as phagogram analysis. The advantage of this approach lies in its ability to rapidly adjust the composition of the medicinal product. However, such flexibility may raise concerns among regulators regarding both quality and clinical aspects of the product, as the drug product may require a variable composition. Several solutions have been proposed to address these challenges, but it is likely that the existing legislative and regulatory framework in the EU will need to be revised to better accommodate this scenario. Notably, the EU institutions are engaged in a substantial revision of the EU pharma legislation^56^, which recognises that certain new categories of medicinal products may require adapted regulations to address their specific characteristics. The EU Commission, the EU Parliament and the Council of the EU have now published their proposals for a Directive of the EU Parliament and of the Council on the Union code pertaining to medicinal products for human use^57–59^, which include provisions to establish an “adapted framework” for “phage-containing medicinal products,” particularly in instance where the composition of the medical product varies based on specific clinical contexts (see Article 28 and Annex VII of the proposals). This proposed framework would entail targeted technical adaptations to the requirements necessary for market authorisation, contingent upon stringent criteria and guided by the scientific input from EMA. As of now, this legislative act remains under discussion and has not yet been adopted.

- **Access to unlicensed products**

In some of the EU Member States, there are established pathways for administering experimental phage therapy to patients with no viable treatment options. These pathways may include compassionate use or participation in clinical trials. In addition, unlicensed medicines may be dispensed to individual patients based on a prescription from a healthcare practitioner. In this case, a pharmacy prepares the medicine according to the applicable pharmacopeia, and it is directly dispensed to the subject (known as the officinal formula route). Another option, known as the named subject use route, allows a medicinal product to be formulated under the prescriber’s responsibility and used directly by the patients. Member States can also temporarily authorise certain medicines under special conditions, which adds to the regulatory complexity. As previously noted, in Belgium, phage preparations must comply with phage monographs for active pharmaceutical ingredients.

While these frameworks enable patients with limited treatment options to access phage therapy, they do not contribute to assessing the efficacy of the treatment. Consequently, such pathways cannot be recognised by the EU regulator for medicine approval. The EMA recommends that all developers engage with the EU regulatory network as early as possible so that the respective programmes can be thoroughly discussed, and advice can be provided.

### Canada (Health Canada)

Addressing the rising threat of AMR is a top health priority for Canada. Guided by the Pan-Canadian Action Plan on AMR (2023–2027) (PCAP), Canada is advancing a One Health, multi-sectoral approach to combat AMR^60^. The PCAP Year 1 Progress Report, published in September 2024, highlights early milestones and ongoing activities across five pillars, including research and innovation^61^. Health Canada’s specific AMR priorities focus on preserving the effectiveness of existing antimicrobials while facilitating access to new treatment^62^. In parallel, Health Canada is modernising its regulatory framework to support innovation within the healthcare system, as outlined in its Regulatory Innovation Agenda^63^. Recognising the importance of international collaboration, Health Canada actively participates in global partnerships to advance both domestic and international AMR objectives, including the International Coalition of Medicines Regulatory Authorities (ICMRA) and the Regulatory Agencies Global Network against AMR (RAGNA).

- **Regulatory considerations**

Health Canada classifies both natural and recombinant phages as biological medicinal products (i.e., drugs) when they are used to treat, mitigate, or prevent disease in humans or animals, as per the *Food and Drugs Act*^64^, Schedule D^65^, and the *Food and Drug Regulations*, Part C, Division 4^66^. Currently, no phage products have received market authorisation in Canada. Therefore, phage therapy is considered an experimental drug in Canada and is only available through a clinical trial that has been reviewed and authorised by Health Canada as per the *Food and Drug Regulations, Part C, Division 5:“Drugs for Clinical Trials Involving Human Subjects*.^67^”

Sponsors wishing to conduct a clinical trial in Canada can submit a clinical trial application (CTA) to Health Canada, who will review the application within 30 days. Health Canada can object to the trial if the requirements in the *Regulations* are not met. Alternatively, if the application meets all standards, a No Objection Letter to the sponsor will be issued, allowing the trial to proceed. Sponsors are encouraged to contact Health Canada’s Biologic and Radiopharmaceutical Drugs Directorate (BRDD)^68^ for specific guidance related to their proposed single-patient or larger phage therapy clinical trials. In addition, it may be beneficial for sponsors to request a meeting with Health Canada when planning their clinical trial application (CTA) in order to ask questions, as needed. Notably, there is no cost to sponsors for these preparatory meetings.

To date, Health Canada has authorised several single-patient clinical trials focused on studying phage therapy for various infections, including recurrent urinary tract infections, cystic fibrosis lung infection, and periprosthetic joint infections that have not responded to traditional antibiotic treatments (unpublished data; see clinicaltrials.gov). It is encouraging to see an increasing interest in the clinical application of phage therapy in Canada. However, Health Canada recognises that more clinical data is required in needed to further demonstrate the efficacy of phage therapy. Clinical trial sponsors, including those from industry, academic hospitals, or other research institutions, are encouraged to consider phage therapy in larger-scale clinical trial settings to advance our understanding of phage biology.

- **Randomised, controlled clinical trials**

Since Health Canada has not yet received clinical trial applications for phage therapy involving more than a single patient, sponsors are strongly encouraged to arrange a pre-CTA meeting with Health Canada as early as possible during the development process. This meeting should focus on discussing clinical, non-clinical, and quality data requirements. Given the extremely diverse nature of phage biology, regulatory requirements may evolve in a risk-based manner, building on what was required in single-patient trials as clinical development progresses. Sponsors can refer to existing Health Canada guidance^69^ for clinical trial sponsors for additional information. Although GMP compliance is not required for RCTs, the expectation for “GMP-like” conditions will increase throughout clinical development. This is essential to ensure consistency in phage manufacturing, quality control, product safety, and robustness of clinical trial results. However, given the innovative and unique nature of phage therapy, regulatory requirements may vary according to the proposed phage product, indication, route of administration, patient population, and trial design.

- **Open label single patient clinical trials**

Single-patient clinical trials are regulated under the same authority as larger clinical trials, specifically under Part C, Division 5 of the *Food and Drug Regulations*. When submitting a CTA for a single-patient study, the sponsor should provide a rationale and supporting information (e.g., literature and clinical data) demonstrating that the patient meets at least one of the following criteria: (1) they have a serious, life-threatening condition, (2) they are not eligible for or exhausted all other treatment options, and (3) there is no other way to access treatment. In addition, the CTA must include information about the investigational product, which should cover dosage form, strength, rationale for dose selection, route of administration, frequency of re-administration, and duration of therapy. It should also include available safety data, potential for efficacy in the patient considered, and identified potential risks. Furthermore, the CTA should contain a detailed treatment and follow-up plan, safety monitoring and risk mitigation measures, evidence of Research Ethics Board approval, and a plan for reporting study outcomes, including safety and efficacy.

In terms of quality data requirements, Health Canada has developed draft guidance (unpublished; available upon request) on key quality attributes that need to be included in the CTA. In urgent situations, such as when a patient has a serious, life-threatening infection, Health Canada will require essential information related to the phage history, lytic activity, potency, impurities, sterility, and endotoxin level, as well as critical manufacturing processes and controls, and storage and transportation. In less urgent situations, Health Canada will require this same information, along with additional phage characterisation and details about manufacturing processes and controls. This additional characterisation should include phage genomic sequencing (for identity), assessment of temperate life-cycle properties (such as integrases, recombinases, transposases), resistance and toxin genes, and stability. This information should be provided for Clinical Trial Material intended for subjects, or for a product derived from the same starting materials (e.g., phage stocks) and manufactured by comparable processes.

While phages used in single-patient trials can be sourced from existing phage banks inside or outside of Canada from well-established research institutions, the Canadian government is currently developing guidelines and phage sharing agreements with various industry and academic partners. The goal is to establish a Canadian government-run phage collection and phage susceptibility testing service^61^.

- **Specific aspects applying to personalised phage products**

There is currently no specific regulatory framework in Canada tailored to phage therapy or personalised phage products. However, the Advanced Therapeutic Products Pathway (ATPathway)^70^ is an agile licensing framework established in 2019 through legislative amendments to Canada’s Food and Drugs Act. This framework allows for flexible regulation of innovative health products. ATPs may include drugs, devices, or combinations thereof that do not fit into Canada’s existing *Food and Drug Regulations* due to their complexity or uniqueness. ATPs could encompass innovative products like personalised phage therapies, which are developed at the point of care and/or manufactured and administered in ways that differ significantly from traditional health products. This framework could enable Health Canada to customise regulatory requirements for phage therapy in the future, ensuring appropriate yet flexible regulatory oversight, while ensuring appropriate standards for safety, efficacy, and product quality. Finally, the ATPathway could facilitate improved access to potentially transformative treatments for Canadian patients, advancing Canada’s priority to combat AMR.

- **Access to unlicensed products**

In Canada, patients may access unlicensed phage products through Open Label Single-Patient Clinical Trials, as detailed in the section above.

### United Kingdom (The medicines and healthcare products regulatory agency)

The UK’s Medicines and Healthcare Products Regulatory Agency (MHRA) is actively working to address the threat of AMR through both national and international efforts. This includes the 5-year AMR National Action Plan, participation in regulatory network AMR activities and initiatives, such as the International Coalition of Medicines Regulatory Authorities (ICMRA) and the Regulatory Agencies Global Network against AMR (RAGNA). The MHRA is also involved in the development and production of reference materials and World Health Organisation (WHO) international standards that are essential for assuring the quality, safety, and efficacy of biological medicines; at the time of writing, there are three phage reference materials/methods in development. The MHRA also has external funding for regulatory science research that supports research and innovation focused on AMR.

UK researchers are significantly interested in the use of phages across the One Health spectrum, and there is an established and well-connected community. In 2023, the UK House of Commons Science, Innovation and Technology Committee (SITC) launched a public enquiry titled ‘The antimicrobial potential of bacteriophages’ following a successful pitch by Professor James Ebdon, Professor of Environmental Microbiology at the University of Brighton, UK, on behalf of Applied Microbiology International^71^. This enquiry considered “over 40 pieces of written evidence and held three oral evidence sessions [hearing from] academic experts and clinicians from the UK and from around the world, funders, Small and Medium-sized enterprises (SMEs), regulators and government officials.” The SITC published the enquiry report in January 2024, which contained several recommendations, to which the government published its response on March 1^st^,2024^72^. The recommendations and responses highlight that although regulatory frameworks for using phage therapy are defined and in place in the UK, successful implementation and adoption require coordinated activities across the entire healthcare ecosystem and clear communication of the relevant processes.

- **Regulatory considerations**

This section covers the legal definition, pathway(s) for authorisation, and regulatory recommendations pertinent to phage medicinal products in the UK. The MHRA published ‘Regulatory considerations for therapeutic use of bacteriophages in the UK' on the 4^th^ June 2025^73^, which provides comprehensive regulatory information pertaining to the development and use of phages to patients, via both licensed and unlicensed routes, including ‘specials’ clinical trials and imports.

In the UK, phages that are isolated or manufactured with the intention of treating a medical condition are classified as biological medicinal products. Consequently, they are subject to the Human Medicines Regulations 2012 (HMRs, as amended). Broadly, phages are classed as biological medicines. If a phage is genetically modified, it may, in certain instances, also be classified as an advanced therapy medicinal product (ATMP), contingent upon the nature of the modification. ATMP is a class of biological medicines subject to both HMRs and separate legislative requirements. For licensed medicinal products, the HMRs outline the criteria for assessing safety, quality, and efficacy to grant a marketing authorisation (license). Currently, there are no licensed phage medicines available in the UK.

For unlicensed medicines, often referred to as “Specials”, Regulation 167 of the HMRs permits the supply of medicines without a marketing authorisation (license) under certain circumstances. This exemption is narrowly defined in the interest of public health since unlicensed medicines have not been evaluated by the licensing authority against the criteria of safety, quality, and efficacy. An unlicensed medicinal product, a “special,” can only be supplied to meet the special clinical needs of individual patients when no available licensed medicinal products can fulfil those needs. Determining whether an individual patient has unique needs that cannot be addressed by a suitably licensed product is a matter for the prescriber responsible for the patient’s care. The prescriber holds the responsibility for the use of unlicensed. An unlicensed medicine may be supplied following a bona fide unsolicited order and is formulated in accordance with the specific requirements of the prescriber.

An unlicensed medicine can either be manufactured in the UK or imported. Manufacturing in the UK must be conducted by the holder of a Manufacturer’s “Specials” license in appropriate facilities, subject to GMP inspection by the MHRA. Importers of unlicensed medicinal products into the UK must hold appropriate licences for such imports. Prior to importing, the importer must notify the MHRA of their intent to import the unlicensed medicine and provide a set of supportive documentation, which will be assessed by the MHRA. The MHRA retains the authority to object to the importation of unlicensed medicine if significant risks are identified that could negatively impact patients. Each import request is assessed on a case-by-case basis, considering both the product and the special clinical needs identified by the prescriber. Medicines imported into the UK as unlicensed medicines are usually required to comply with similar GMP expectations as those manufactured domestically.

Section 10 of the Medicines Act 1968 and Regulation 4 of the HMRs provide an exemption from the restrictions imposed by regulations 17(1) (manufacturing of medicinal products) and 46 (requirement for authorisation) of the HMRs for “anything which is done in a registered pharmacy, a hospital, a care home service or a health centre and is done there by or under the supervision of a pharmacist and consists of preparing or dispensing a medicinal product in accordance with a prescription given by an appropriate practitioner, or assembling a medicinal product.”^74^ The conditions for extemporaneous preparations are not determined by the MHRA, so consultation with appropriate experts is recommended. The General Pharmaceutical Council regulates pharmacists, pharmacy technicians, and pharmacies in Great Britain.

The composition of phage ‘cocktail’ products may require adjustment or updating over time to maintain their effectiveness against circulating strains. Such product variations may be a suitable route to making these changes. Evidence will need to be provided to the competent authority to demonstrate that replacing one phage with another in a given cocktail product retains host range and efficacy for the indication for which the product is licensed without additional risk to the product’s quality or safety. Whether a new clinical trial is necessary will depend on the scientific data accompanying the change, and it is advisable to engage in discussion with the regulator. Developers can seek advice from the MHRA^75^, which is currently revising its clinical trial approval process^76^ following the Lord O’Shaughnessy review^77^, employing a risk-proportionate approach. Engaging with the MHRA through its Innovation Office^78^ for advice is highly advisable.

Regulatory oversight for genetically modified organisms, including genetically modified phages, is administered by either the Health and Safety Executive (HSE) or the Department for Environment, Food & Rural Affairs (Defra), depending on the nature of their release. It is also crucial for product developers intending to introduce products to markets to engage with procurers and/or health technology assessment (HTA) bodies. In the UK, the National Institute for Health and Care Excellence (NICE) and NHS England launched a new subscription model. This model acknowledges that investing in antimicrobials is often not commercially attractive due to cost expectations and limited usage; it decouples the value added from the number of prescriptions sold. Currently, the subscription model^79^ only encompasses traditional (small molecule) antimicrobials, but eligibility criteria are expected to expand to include non-traditional therapies, such as phage and microbiome treatment, which is reviewed regularly.

For product developers seeking licensure in the UK, it is advisable to understand evidence requirements and evaluation processes. NICE offers a specific service called NICE Advice^80^ to support companies seeking to enter the NHS market.

- **Specific Aspects Applying to Personalised Phage Products**

Personalised phage therapies may be classified as either licensed or unlicensed medicinal products. Other types of licensed personalised therapies include engineered cell therapies and mRNA cancer immunotherapies. It is envisaged that personalised phage therapy could follow a similar licensing route, and, like cell and mRNA therapies, be subject to GMP. Although the ‘complexity’ of phages is often cited as a barrier to GMP compliance, it is important to note that individualised cell and gene therapies, which are prepared and administered under GMP, have been adopted by the NHS and are recommended by NICE for several adult and paediatric indications. Guidance on pharmacy institutional readiness for these products^81^ has been developed to facilitate adoption across the NHS.

- **Access to unlicensed products – challenges and considerations**

Beyond the role of MHRA in regulating unlicensed medicinal products, governance for the use of unlicensed medicines is the responsibility of individual NHS Trusts. It is imperative for individuals seeking to utilise therapeutic phages to recognise that decisions regarding the use of unlicensed medicines are determined based on local unlicensed medicines policies within NHS Trusts, not by the MHRA.

Unlicensed medicines manufactured in the UK must adhere to GMP standards. Unlicensed medicinal products imported for use in the UK undergo quality evaluation by the MHRA Imports Office to ensure they meet UK quality requirements. To date, imported phages have been used in the UK on an unlicensed basis, with some cases included among the 100 reported cases from Belgium in 2024^8^.

### United States (food and drug administration)

Phages proposed for the treatment of infectious diseases in humans are regulated as biological drug products by the Office of Vaccines Research & Review (OVRR) at the Centre for Biologics Evaluation and Research (CBER), within the United States Food and Drug Administration (US FDA). Clinical investigations of unlicensed products or unapproved uses of licensed products intended to cure, mitigate, treat, or prevent a disease require an Investigational New Drug (IND) application. This requirement applies regardless of whether the product is intended for commercial development or for expanded access (compassionate use).

- **Regulatory considerations**

For considerations of when to approach the FDA for potential use of phage in the clinic, sponsors should first identify a specific, attainable investigational product and develop a draft clinical plan or synopsis. At that point, it is appropriate to request a pre-IND meeting with OVRR to obtain input on the proposed development plan and other pertinent, product-specific regulatory questions. Guidance on this aspect of the product development process can be found in Formal Meetings with Sponsor and Industry^43^. This formal feedback from the FDA should be carefully considered when a sponsor is preparing to submit the IND application.

The IND application should include product-specific information on chemistry, manufacturing, and controls (CMC), the clinical plan, and any non-clinical information such as proof-of-concept studies that may inform clinical development (21 CFR 312). The submission should articulate a rationale for the clinical studies and product development^82^. Generally, an Investigator’s Brochure should also be included^83^. The information outlined below does not encompass a complete list of what may be required for an IND submission. Each file is reviewed on a case-by-case basis to enable the Agency to assess risk/benefit and help to ensure the safety of study participants.

- **Chemistry, manufacturing and controls (CMC)**

It is essential to demonstrate that the investigational phage product is manufactured in a manner appropriate for the stage of clinical development. While phage preparations must be safe for human administration at all phases of development, full GMPs are generally not required for early phase studies^84,85^. The phage product must be clearly defined. It can be a single phage or a combination of several phages, known as a ‘cocktail’ that targets one bacterial strain or multiple bacterial strains. In addition, it can be a fixed cocktail or a personalised product comprised of engineered or naturally occurring phage. The phage product and the propagating strain should be fully characterised, including identity (annotated sequencing and phenotyping), sterility, and characterisation of any deleterious genes, mobile elements, prophage, and exotoxins. The history and sourcing of the phage should be included. Plans for cell banking and relevant data should also be included. Data regarding the drug substance and drug product should be evaluated and provided. Proposed investigational product information on potency (Plaque Forming Units (PFU)/ml), transduction (based on the presence of 16S rDNA of the bacteria that is used to proliferate phages), demonstration of removal of residuals/contaminants, phenotyping, sterility, pH, appearance, endotoxin and exotoxin, and host cell proteins (HCP) should be included. In addition, information about any diluent and placebo should be provided. A stability plan with proposed expiry dating and any relevant supporting data should be included. If sponsors are planning a personalised/individualised approach to their phage therapy clinical trial, the same information must be submitted for each phage strain used (as well as propagating bacterial strain and history of phage) prior to treatment.

 The FDA recommends providing a description of the manufacturing process, along with a flow diagram and accompanying narrative. Manufacturing facility information, including any other products handled or manufactured at the site, should be included^86^. Information on dedicated or shared equipment used at the manufacturing site should be supplied. A complete list of raw materials and sources of each must also be included.

In the instance where the IND sponsor may not manufacture the product and therefore may not have access to CMC information, the sponsor must include a cross-reference to the manufacturing information of the product in a Type II CMC master file (MF). This allows for the submission of confidential information and data that is reviewed in the context of a specific IND. This MF must be submitted to CBER, and the MF holder must provide a Letter of Authorisation (LOA) to the IND sponsor to allow the IND to cross-reference the MF. An MF can be used to support multiple INDs. It is important to note that Type II MFs can only be used during the IND development stage^87^.

- **Early clinical studies (Safety)**

The FDA’s OVRR/CBER reviews Phage INDs for infectious disease indications. For early clinical studies, the information discussed below is recommended to be included. The route of administration of the investigation product can include, but is not limited to, intravenous (IV), intraperitoneal (IP), nebulised, topical/direct, intravascular, and intraarticular. This may or may not involve the use of a device. If sponsors are using a device to administer the phage product, the device information should be submitted with the IND. It is also essential to provide details on where the clinical trials will be conducted, including other investigators and sub-investigators, as known and as appropriate (21 CFR 312).

- **Design**: The FDA has numerous disease-specific guidance documents pertaining to clinical trial design. It is recommended that sponsors refer to these Guidance Documents when designing their studies, as applicable and appropriate^88^. It is also recommended that the clinical trial design build in testing and matching of the infecting bacterial strains of the trial participants against the investigational phage strain, including collecting data on any developed resistance of the bacteria to the phage. Collecting this information during development may inform future trial design.
- **Collection of laboratory measurements:** Baseline measurements and assessments at critical points during the trial are important. If the investigational phage product is administered via IV or other invasive means, liver function testing should be included. This information and scheduling of events can be presented in tabular format. Investigational product route of administration, frequency of dosing, and length of treatment must be prospectively defined, with proposed follow-up time. Study-wide and individual pausing rules must be proposed, with relevant toxicity grading scales provided. The safety and rights of the participants must be assured^89^. The clinical study design should incorporate a prospectively defined decision tree if phage strains may be proposed to be switched out, including culture results that are critical for these decisions (such as for developed resistance or futility), or phage administration extended. It is noted that any modifications and changes to the phage strains used during a clinical trial or in development may confound data interpretation and affect clinical trial design.
- **Objectives and endpoints**: Primary objectives for early clinical development are generally safety and tolerability. Randomisation and blinding are not always mandatory in early (phase 1) development stages. Consult with the FDA if patient-reported outcomes are planned to be used in the clinical assessment^90^. Determining an appropriate case definition is important for the disease studied.
- **Participant screening**: Defining the target population and establishing criteria for enrolling participants are crucial. In addition, the following factors should be considered: types of infections, locations of infections, whether there are single or multiple infecting bacterial strains, prior medical treatments, allowable use of concomitant use of medications, and recommended standard of care for the specific disease. A collection of relevant infection history (culture results) of the participant and treatments previously used is also recommended.
- **Statistics**: Statistical analyses used in early studies are generally descriptive rather than statistically powered. Information on methods to define and demonstrate safety and any preliminary efficacy must be included, along with measurable objectives. The protocol and a statistical plan (if included) should define the ‘intent to treat’ population and outline how missing data will be handled.

- **Consistency of information**

Information submitted to the IND should be focused specifically on the product proposed for clinical use. All information should be consistent and accurate throughout the IND, including the protocol (schedules, charts, narratives), Investigator Brochure (IB –ICH E6 (R2) Good Clinical Practice, part 7 or FDA publication), the general investigational plan, and the summaries of clinical, nonclinical, and product information^83^.

- **Expanded Access/Compassionate Use for single patients**

Single-patient, Expanded Access (EA) Investigational New Drug applications (SPINDs), also known as Compassionate Use, may be requested for FDA review when a patient has a serious or immediately life-threatening disease with no other treatment options available. The SPIND should be directed to OVRR/CBER within the FDA. Specific criteria must be met for consideration of expanded access as described in INDs 21 CFR 312.305 (a) (1–3) and 21 CFR 312.310. In summary, they are:

- Patient has a serious or immediately life-threatening disease or condition.
- There is no comparable or satisfactory alternative therapy to diagnose, monitor, or treat the disease or condition.
- Patient enrolment in a clinical trial is not possible.
- Potential patient benefit justifies the potential risks of treatment.
- Providing the investigational medical product will not interfere with investigational trials that could support a medical product’s development or marketing approval for the treatment indication.

Generally, the types of CMC and clinical information provided for controlled clinical trials can be applied to SPINDs, although the level of detail may be significantly reduced. In most cases, a treating physician submits the SPIND, which includes the treatment plan and patient history, while a phage manufacturer may supply the CMC information. This may also be provided from one source, but this information must be made available for review. The FDA has a comprehensive website that should be consulted^91,92^

## Conclusion

**Fig. 1:**
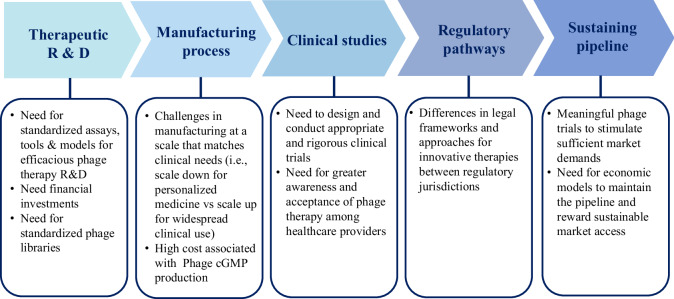
Challenges in advancing phage therapy are categorised into five areas, each with prioritised needs listed. R&D Research & Development, cGMP Current Good Manufacturing Practices.

Despite over a dozen ongoing phase 1–3 clinical trials, the field continues to face significant obstacles in producing the data required for demonstrating the value of phage therapies. Several scientific and regulatory obstacles may deter developers and investors from further engaging in phage therapeutic endeavours (Fig. 1), including:

- The absence of standardised assays and tools for testing for phage susceptibility, which complicates the establishment of reliable susceptibility criteria and the development of personalised approaches^41,93^;
- A need for effective animal models and experimental tools that accurately reflect phages-bacteria interactions and phage distribution within hosts, to facilitate the development of appropriate PK/PD models and the determination of optimal dosage regimens^32^;
- Regulatory aspects concerning personalised phage therapy, including a lack of clearly defined criteria for approving personalised phage therapy medicinal products, and existing or perceived challenges associated with current GMP requirements;
- Logistical difficulties in ensuring broad and sustainable access to personalised therapies; and
- Uncertainty surrounding the potential market for phage products raising concerns about possible market failure.

Beyond the efforts needed to address these challenges, TATFAR members identified three areas requiring additional efforts and collaboration. The first area involves exploring novel therapeutic options, such as combination therapies involving antibiotics and phage products. Recent studies have shown promising results by examining both synergistic and antagonistic interactions among different phages, as well as employing combinations of phages and antibiotics to tackle infections caused by highly threatened pathogens^94–97^. This research underscores the potential for innovative treatments, which require further exploration to advance rigorously in clinical and regulatory settings. The TATFAR framework could significantly facilitate this advancement. The second area emphasises the importance of advocating for a One Health approach in the development of phage therapy^98^. Integrating insights from various sectors enhances the understanding of how phage therapy can be safely and effectively applied across different domains and facilitates the development of holistic policies. Finally, there is a need to further explore the applicability of phage therapy in resource-limited settings, particularly in low- and middle-income countries (LMICs). Several experts suggest that phage applications could be highly beneficial in LMIC^99^ due to the relative simplicity of isolating phages from sources such as hospital waste or sewage water, utilising readily available tools. In addition, phage products can potentially be developed and manufactured quickly and cost-effectively, with the advantage of being formulated as dry powders that do not require refrigeration for storage. Nevertheless, significant efforts are necessary to facilitate the widespread adoption of phages in LMICs, contingent upon confirming their efficacy and safety. This involves establishing suitable regulatory processes, fostering commercial incentives, and securing cultural acceptance.

To advance phage therapy effectively, a robust dialogue among stakeholders, including scientists, policymakers, regulators, practitioners, and patients, is essential to identify and overcome the existing or perceived scientific and regulatory challenges. The clinical benefits of phage therapy, whether delivered as currently unlicensed personalised preparations or fixed phage products, remain uncertain pending validation from ongoing and upcoming clinical studies. Nevertheless, given the increasing burden of AMR on public health and the scarcity of new antibiotics, further exploration of this therapeutic approach is both necessary and justified.

The in-person meeting of TATFAR members in November 2023 provided a valuable platform to strengthen existing collaborations and to engage in discussions on the regulatory aspects of phage therapy. These inaugural discussions have propelled progress in this important field, emphasising the necessity of international collaboration to address shared priorities in combating AMR. Although primarily involving TATFAR member countries, stakeholders from Far Eastern Asian countries, including Japan, Singapore, South Korea, and Taiwan, were invited to foster future dialogues across continents. This aligns with TATFAR’s mission to open further opportunities for phage therapy from both scientific and regulatory perspectives on a global scale.

A notable outcome of these efforts was two recent research announcements among TATFAR members. Firstly, the US NIH announced a phage-specific Notice of Funding Opportunity (NOFO) for establishing Centres for Accelerating Phage Therapy to Combat ESKAPE Pathogens (CAPT-CEP)^100^. This initiative solicited applications from the global phage research community. These centres will focus on developing appropriate preclinical assays, tools, and models for robust phage therapy research and development (R&D) to address identified challenges in phage trials (Fig. 1). By bridging gaps in current technology, the ultimate goal is to facilitate the development of efficacious phage products and advance them to rigorous testing and use in clinical settings. Secondly, Horizon Europe 2025, commissioned by the EC, has announced support for clinical trials to test the safety and efficacy of phage therapy for treating antibiotic-resistant bacterial infections^101^, underscoring the importance of continued support for the phage therapy field among the TATFAR members.

As demonstrated in this perspective, TATFAR countries are actively engaged in initiatives to support the development of phage therapy and to expand access to such therapy, albeit with different perspectives and approaches. TATFAR members will continue an open dialogue on phage therapy clinical trials, regulatory requirements for phage therapy in their respective countries, research opportunities to address shortcomings in current technologies, and phage use within the One Health approach. As the field of phage therapy continues to evolve, challenges are expected to be addressed through ongoing R&D, international collaboration, and regulatory developments. Continued dialogue within TATFAR and with external partners, including LMICs, could be significantly beneficial in overcoming these obstacles.
